# The Galvanic Corrosion of AM60B Coupled with DC01 in Simulated Environments with Varying Water Salinity

**DOI:** 10.3390/ma17164099

**Published:** 2024-08-19

**Authors:** Wei Zhang, Chuntang Yu, Wanqi Pu, Xiaoyun Li, Chi Zhang, Renju Cheng, Guozheng Quan, Linlin Yang, Fuhui Wang

**Affiliations:** 1 School of Materials Science and Engineering, Chongqing University of Technology, Chongqing 401320, China; 2 School of Materials Science and Engineering, Chongqing University, Chongqing 400044, China; 3 The Sword of the Spirit of Beijing Science and Technology Co., Ltd., Beijing 100080, China; 4 Shenyang National Laboratory for Materials Science, Northeastern University, Shenyang 110819, China

**Keywords:** magnesium, AM60B, galvanic corrosion, EIS, FEM

## Abstract

The galvanic corrosion performance of AM60B coupled to DC01 was characterized in simulated environments with varying water salinity. The results showed that the coupled DC01 effectively accelerated the corrosion rate of AM60B, and the increased salt concentration had a significant effect on the deterioration process. The corrosion of AM60B mainly exhibits metal dissolution, and the formed Mg(OH)_2_ has weak a protective effect on the alloy substrate. Furthermore, the distributions of the corrosion potential and the corrosion current density of the AM60B/DC01 couple were simulated and intensively discussed.

## 1. Introduction

Magnesium (Mg) and its alloys, as the lightest metal structural materials, have attracted wide interest for application in aviation, aerospace, automotive, and other industries, owing to their low densities, high damping capacity, good casting properties, and ease of recycling [1,2,3,4,5,6]. The principal drawback of magnesium alloys is their poor corrosion resistance; especially, the corrosion rate of magnesium alloys is too high to use in environments containing aggressive electrolytes [5,6,7,8,9,10,11,12]. Direct contact between the magnesium alloys and heterogeneous materials (e.g., steel, aluminum alloys, and carbon composite materials) is unavoidable when constructing hybrid structures in conducting-medium environments, and galvanic corrosion is liable to occur, thereby leading to a premature structural failure [11,12,13,14,15].

The typical lightweight material, AM60B magnesium alloy, connected with DC01 has been widely applied in an amphibious vehicle [16,17]. It can effectively reduce the weight of the whole vehicle and improve the overall efficiency of the body, which has great potential for application and promotion [18,19]. There are concerns about the poor corrosion resistance of the magnesium alloy and the heterogeneous connection state; damage to the body structure is possible, and service safety is still lacking, especially in offshore, freshwater, and other environments involving the erosion of different concentrations of chloride ions [20,21]. The service safety of this type of structure is still lacking, so it is necessary to clarify the corrosion resistance of the AM60B/DC01 hybrid structure in this type of environment to improve its reliability.

In the current work, the corrosion behavior of AM60B coupled and uncoupled to DC01 is studied in 0.1, 0.5, and 3.5 wt. % NaCl solutions to simulate various water salinity environments. The corrosion resistance performance is evaluated by SEM, electrochemical test, and FEM simulation. And the effect of salt concentrations on the galvanic corrosion behavior in the AM60B/DC01 structure is analyzed, and the related mechanism is intensively discussed.

## 2. Experimental Methods

### 2.1. Material and Specimen Preparation

The commercial AM60B magnesium alloy and DC01 low-carbon steel were used as the tested materials in this study. The AM60B compositions (wt. %) were 5.5-Al, 0.15-Zn, 0.5-Mn, 0.0015-Ce, 0.005-Th, 0.005-Cd, and balance Mg. The DC01 consisted of 0.12-C, 0.60-Mn, 0.045-P, 0.045-S, and balance Fe (all in wt. %).

The AM60B and DC01 samples were both cut into specimens with dimensions of 10 mm × 10 mm × 3 mm. A copper wire was connected to the back of each sample, and they were secured using epoxy resin, ensuring that a 1 cm^2^ working surface was exposed. After the epoxy resin solidified, the working surfaces were abraded using silicon carbide paper up to 2000 grit. Any oil residues on the working surface were removed using anhydrous ethanol, followed by thorough cleaning with deionized water and drying.

### 2.2. Microstructure Characterization

The surface morphologies of the sample before and after the coupling test were observed by tungsten scanning electron microscopy (TFSEM, JCM-7000 NeoScope, JEOL, Tokyo, Japan) equipped with an EDS probe, and the corresponding elemental distributions and compositions were characterized by the self-contained EDS probe to evaluate the corrosion degree and status.

### 2.3. Electrochemical Measurements

All the electrochemical tests were conducted at a room temperature of 25 °C. The test solution environment consisted of 0.1 wt. %, 0.5 wt. %, and 3.5 wt. % NaCl solutions. The electrochemical workstation used was the Gamry 3000 system from Gantry Company (San Francisco, CA, USA). The typical three-electrode system was employed for the tests. A saturated calomel electrode (SCE) was used as the reference electrode; a platinum electrode with dimensions of 20 mm × 20 mm served as the counter electrode; and the sample acted as the working electrode connected to the electrochemical workstation interface via a copper wire. To obtain accurate data, the sample was stabilized at the open-circuit potential (OCP) for 15 min before any electrochemical test was performed.

The electrochemical behavior of the alloy in the environment was analyzed using electrochemical impedance spectroscopy (EIS). The EIS experiments were conducted in the frequency range of 10^−2^ Hz to 10^5^ Hz with a perturbation of 5 mV. The Tafel extrapolation method was used to calculate the corrosion current density (I_corr_) from the potentiodynamic polarization curves. The scan rate for the potentiodynamic polarization curves was set to 0.3333 mV/s, with the initial potential being the OCP minus 0.25 V and the final potential being the OCP plus 0.25 V. To ensure repeatability of the experiment, each test was repeated three times.

### 2.4. Galvanic Immersion Test

The corrosion potential and current density changes over time for the coupled AM60B and DC01 samples in solutions with different salt concentrations were measured using the CST500 electrochemical noise detector from the CorrSpec company (Singapore). The anode working electrode was AM60B; the cathode working electrode was DC01; and the reference electrode was an SCE. The tests were conducted for a duration of 3 h at a temperature of 25 °C. Each experiment was repeated three times to ensure experimental repeatability.

### 2.5. Modeling Methodology

Simulations were performed to calculate the potential and the current density distribution during galvanic corrosion; the COMSOL v6.0 Multiphysics software was adopted to describe the corrosion processes. All physicochemical processes considered in this model included the following three categories: electrochemical reactions, mass transport (diffusion and migration), and homogeneous reactions (hydrolysis and complex formation).

## 3. Results

### 3.1. Initial Microstructure and Elemental Distribution of the Coupling Materials

**Figure 1:**
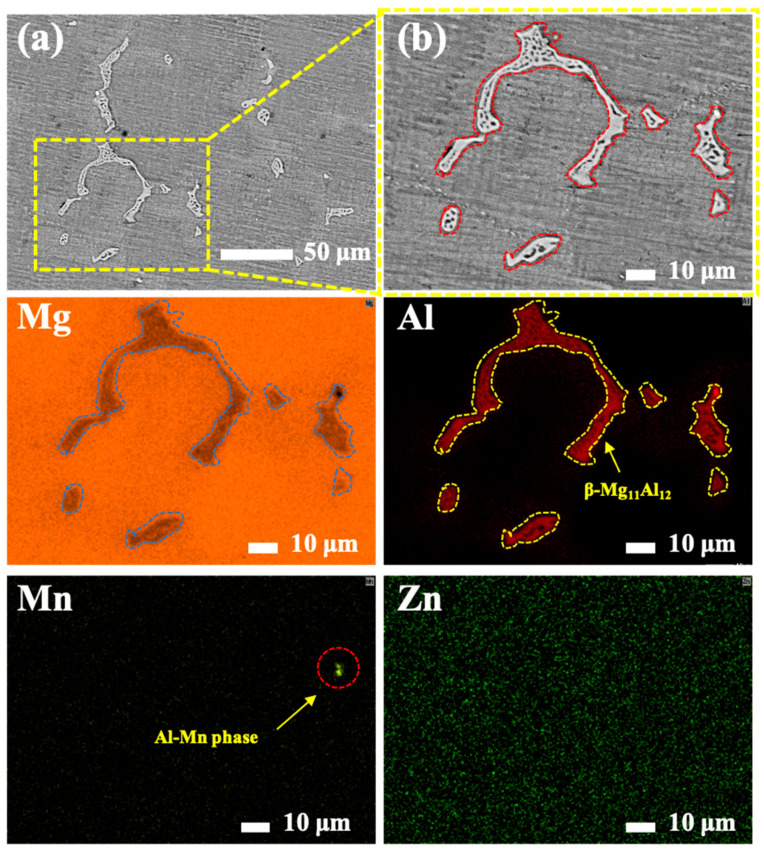
Surface morphology of as-polished AM60B (**a**), the magnified view (**b**) and corresponding element distribution map.

Figure 1 shows the surface morphology and corresponding element distribution of the as-polished AM60B. It can be observed that large amounts of white reticular and discrete point precipitates were dispersed throughout the magnesium matrix (as seen in the BEI image), and the corresponding element distribution (especially for Mg and Al) exhibited that white second precipitate was poor in Mg and rich in Al. This phenomenon implied that the white β-Mg_11_Al_12_ s phases were precipitated in an α-Mg matrix during the casting preparation processes.

**Figure 2:**
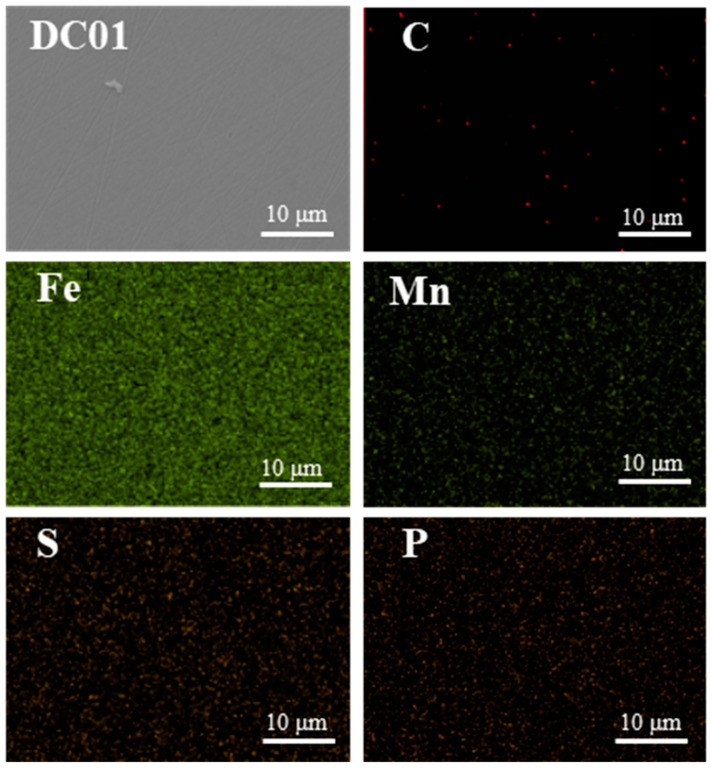
SEM images and corresponding elemental distribution on as-polished DC01.

Figure 2 shows the surface morphology and the corresponding element distribution of the as-polished DC01. It can be seen that the surface of the DC01 was relatively flat and exhibited a ferrite-dominated microstructure with tiny dark particles, which is attributed to the precipitation of carbides.

### 3.2. Electrochemical Measurements of the Coupling Materials

**Figure 3:**
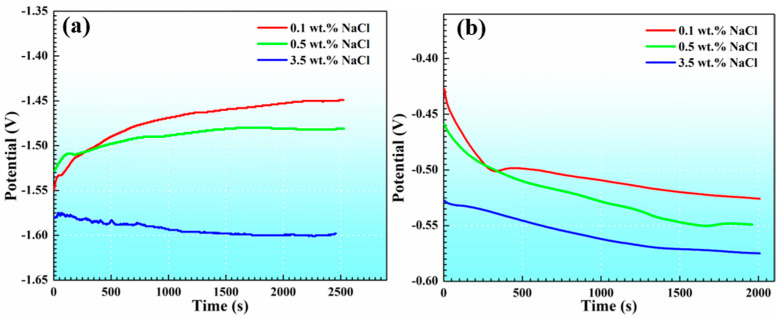
Open-circuit potentials of AM60B (**a**) and DC01 (**b**) in solutions with 0.1, 0.5, and 3.5 wt. % NaCl concentrations.

Figure 3 shows the open-circuit potential curves of AM60B and DC01 in NaCl solutions with concentrations of 0.1, 0.5, and 3.5 wt. %, respectively. The stable open-circuit potential of AM60B was −1.45 V, −1.48 V, and −1.60 V in 0.1, 0.5, and 3.5 wt. % NaCl solutions, respectively. While the stable open-circuit potential of DC01 was −0.53 V, −0.55 V, and −0.57 V in 0.1, 0.5, and 3.5 wt. % NaCl solutions, respectively. AM60B is more likely to act as the anode and DC01 as the cathode during the AM60B-DC01 coupling, with a potential difference of about 1 V between the two electrodes.

**Figure 4:**
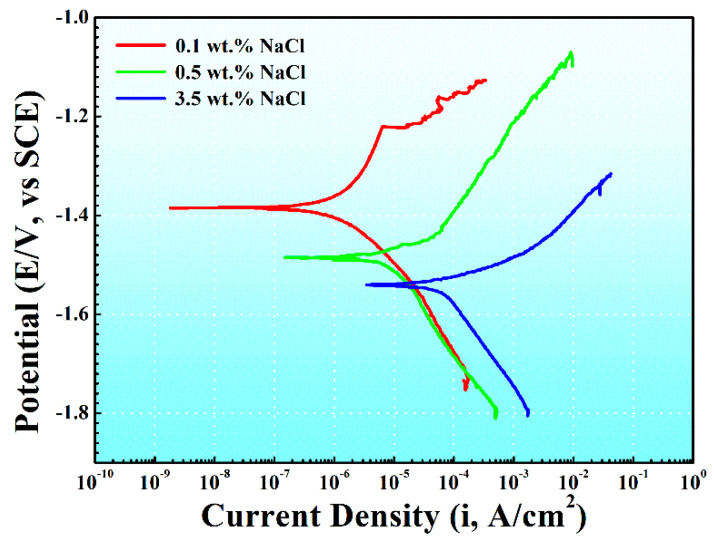
Potentiodynamic polarization curves for AM60B in 0.1 wt. %, 0.5 wt. %, and 3.5 wt. % NaCl solutions.

Figure 4 depicts the potentiodynamic polarization curves of AM60B in solutions with varying NaCl concentrations. The anodic segment of the polarization curve is associated with the dissolution of the magnesium alloy, while the cathodic segment corresponds to the hydrogen evolution reaction. As the NaCl concentration increased, the corrosion current density (I_corr_) gradually increased, and the corrosion potential (E_corr_) gradually shifted negatively. The decrease in the corrosion potential and the increase in the corrosion current density indicated that the corrosion tendency of the magnesium alloy increased and the corrosion rate increased. The corrosion current density (I_corr_) was determined using the Tafel extrapolation method, and the obtained data are summarized in Table 1.

In Figure 4, it can be observed that a pseudo-passivation phenomenon was observed in the anodic branch of the magnesium alloy polarization curve under the condition of 0.1 wt. % NaCl concentration, while it is not observed under other conditions. Pseudo-passivation leads to the formation of a stable oxide layer on the metal surface, thereby reducing the corrosion rate of the metal. However, it is accepted that pseudo-passivation is different from true passivation as it does not completely isolate the metal from the solution. Moreover, the formation of a pseudo-passivation layer is highly dependent on specific conditions, primarily temperature and salt concentration. In our current experiments, a constant room temperature of 25 °C was maintained. When the NaCl concentration exceeds 0.1 wt. %, the balance between the formation and disruption of the pseudo-passivation film is disturbed, resulting in the disappearance of the pseudo-passivation phenomenon.

**Figure 5:**
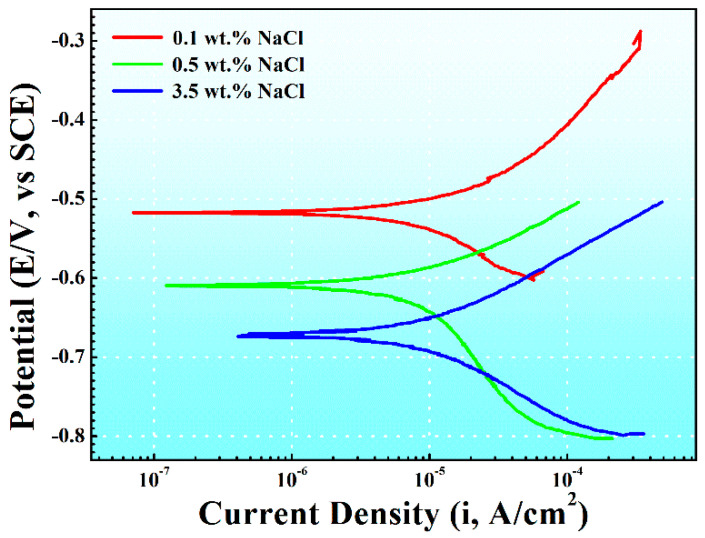
Potentiodynamic polarization curves for DC01 in 0.1 wt. %, 0.5 wt. %, and 3.5 wt. % NaCl solutions.

Figure 5 presents the potentiodynamic polarization curves of DC01 in 0.1, 0.5, and 3.5 wt. % NaCl solutions. The analyzed parameters are summarized in Table 2. In Table 2, the corrosion potential (E_corr_) of DC01 gradually shifts to more negative values with the NaCl concentration increased (from −0.52 V to −0.67 V). The corrosion current density (I_corr_) also increases progressively with NaCl concentration increased (from 6.59 μA/cm^2^ to 9.13 μA/cm^2^), indicating an increase in the corrosion rate as NaCl concentration increases.

In Figure 5, it can be observed that the anodic and cathodic branches of the polarization curves in solutions with different concentrations of NaCl do not exhibit any significant changes in shape. This indicates that the corrosion type of DC01 remains unchanged in solutions with different NaCl concentrations.

**Figure 6:**
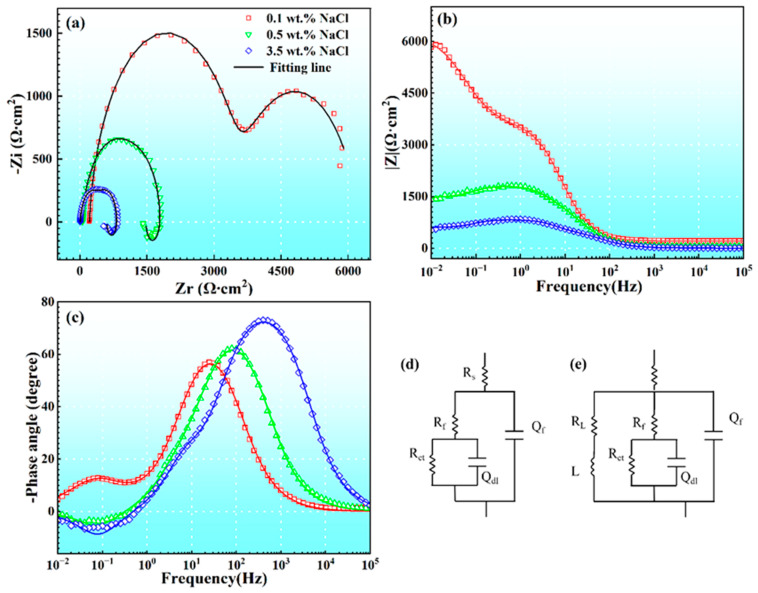
EIS plots of AM60B immersed in various NaCl solutions: (**a**) Nyquist plots; (**b**) Bode impedance magnitude plots; (**c**) bode plots of phase angle vs. frequency, and the corresponding equivalent circuit modes (**d**,**e**).

Electrochemical impedance spectroscopy (EIS) can be utilized to analyze the corrosion characteristics of electrode systems. Figure 6 presents the Nyquist and Bode plots of AM60B in NaCl solutions with different concentrations. The Bode plot consists of two graphs depicting the impedance–frequency relationship and the phase angle–frequency relationship. As shown in Figure 6a, the Nyquist plot exhibited two capacitive loops, one at low frequency and the other at medium and high frequency under the condition of 0.1 wt. % NaCl concentration. When the NaCl concentration increased to 0.5 wt. % and 3.5 wt. %, the Nyquist plot displayed a capacitive loop at medium and high frequency and an inductive loop at low frequency. As the NaCl concentration increased, the diameter and modulus of the capacitive loop in the Nyquist curve gradually decreased.

In Figure 6b, the impedance of 0.1 wt. % NaCl is higher than that of the other two concentrations at all frequencies. These results indicated that the corrosion resistance follows the order of 0.1 wt. % > 0.5 wt. % > 3.5 wt. %. This trend was also evident in the Bode phase angle plot (Figure 6c), in which the peak value of the phase angle gradually shifted to higher frequencies from 0.1 wt. % to 3.5 wt. %. Additionally, the appearance of the inductive loop is often associated with the occurrence of adsorption, desorption, and high corrosion-prone phenomena on the electrode surface [22]. In Figure 6c, it is observed that the phase angle is positive in the low-frequency range under the conditions of 0.5 and 3.5 wt. % NaCl. This indicates that there is a phenomenon in which the voltage phase angle is ahead of the current phase angle. This further corroborates the occurrence of inductance at higher NaCl concentrations. Conversely, the phase angle remained negative at all the test frequencies under the 0.1 wt. % NaCl condition, indicative of a capacitive response. This suggested that there were differences in the corrosion characteristics of the electrode system between the 0.1 wt. % condition and the other higher concentrations. Therefore, two equivalent circuits (Figure 6d,e) were used to fit the 0.1 wt. % (Figure 6d) and the other higher concentration conditions (Figure 6e) [22]. The fitted parameters obtained from the fitting are summarized in Table 3. The equivalent circuit can be represented by Equations (1) and (2), respectively.

For 0.1 wt. % NaCl,

(1) $$\left|Z\right|=Z_{R_{s}}+{\left(\frac{1}{Z_{Q_{f}}}+\frac{1}{Z_{R_{f}}+{\left(\frac{1}{Z_{R_{ct}}}+\frac{1}{Z_{Q_{dl}}}\right)}^{-1}}\right)}^{-1}$$

For 0.5 and 3.5 wt. % NaCl,

(2) $$\left|Z\right|=Z_{R_{s}}+{\left(\frac{1}{Z_{Q_{f}}}+\frac{1}{Z_{L}+Z_{R_{L}}}+\frac{1}{Z_{R_{f}}+{\left(\frac{1}{Z_{R_{ct}}}+\frac{1}{Z_{Q_{dl}}}\right)}^{-1}}\right)}^{-1}\;$$

In the equivalent circuit diagrams, R_s_ represents the solution resistance; R_f_ and Q_f_ represent the film resistance and capacitance over the magnesium alloy surface; R_ct_ represents the charge transfer resistance; Q_dl_ represents the double-layer capacitance; and R_L_ and L represent the resistance and inductance of the inductive branch, respectively. When the NaCl concentration increases, the corrosion products on the surface of the magnesium alloy also increase. However, it is noteworthy that the equivalent circuit parameter R_f_, representing the surface film, does not exhibit a corresponding increase with the rising NaCl concentration, as observed in Table 3. The underlying reason for this phenomenon can be attributed to the following factors: A pseudo-passivation phenomenon occurs on the surface of the magnesium alloy under the conditions of 0.1 wt. % NaCl. The magnesium alloy surface remains intact, and a uniform passivation film covers the magnesium alloy surface. This passivation film acts as a barrier against corrosion. Consequently, R_f_ retains a relatively high value under 0.1 wt. % NaCl conditions. Conversely, the conditions for pseudo-passivation on the magnesium alloy surface are disrupted, leading to the loss of the protective effect of passivation under the conditions of 0.5 and 3.5 wt. % NaCl. The increase in NaCl concentration exacerbates corrosion, resulting in the increased evolution of hydrogen (H_2_) and the loosening of corrosion products on the magnesium alloy surface. These factors collectively contribute to the observed decrease in the value of R_f_ under the 0.5 and 3.5 wt. % NaCl conditions.

**Figure 7:**
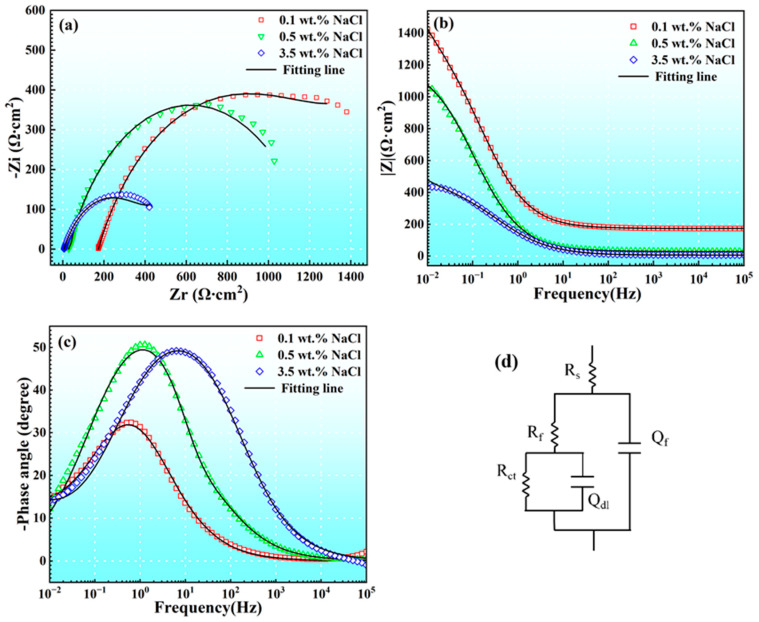
EIS plots of DC01 immersed in various NaCl solutions: (**a**) Nyquist plots; (**b**) Bode impedance magnitude plots; (**c**) bode plots of phase angle vs. frequency, and the corresponding equivalent circuit modes (**d**).

Figure 7 illustrates the electrochemical impedance spectroscopy (EIS) measurements of DC01 in 0.1 wt. %, 0.5 wt. %, and 3.5 wt. % NaCl solutions. Figure 7a represents the Nyquist plot, while Figure 7b,c depict the impedance and phase angle plots of the Bode diagram, respectively. The equivalent circuit used for fitting is shown in Figure 7d, and the fitted parameters are summarized in Table 4 [23]. The equivalent circuit can also be expressed by Equation (1). In the equivalent circuit diagram, R_s_ represents the solution resistance; R_f_ and Q_f_ denote the film resistance and capacitance over the alloy surface; R_ct_ represents the charge transfer resistance; and Q_dl_ represents the double-layer capacitance. Based on Figure 7a, all the three NaCl concentrations exhibited a single capacitive loop in the Nyquist curves, and the diameter and modulus of the capacitive loop decreased as the NaCl concentration increased. In Figure 7b, the impedance value was highest under the 0.1 wt. % condition. These results indicate that the corrosion resistance of DC01 decreases gradually with an increased NaCl concentration, but the corrosion characteristics of the electrode system remain unchanged.

**Figure 8:**
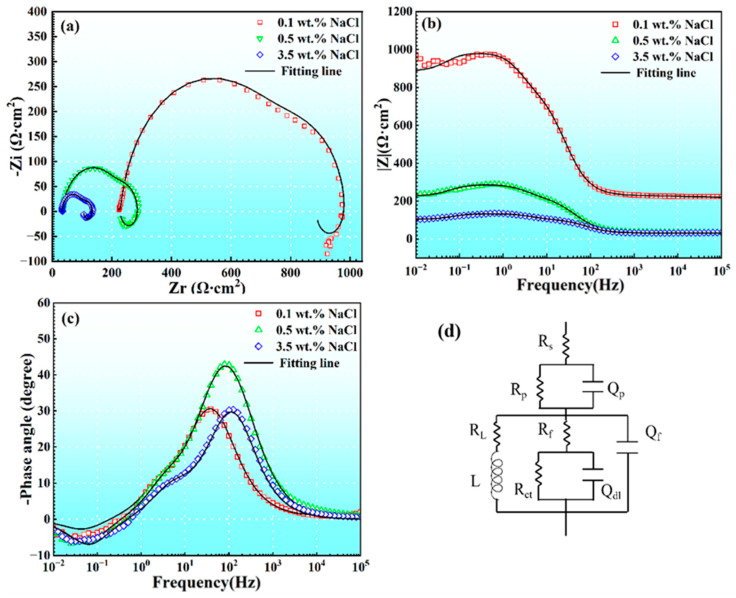
EIS plots of AM60B immersed in various NaCl solutions after coupling for three hours. (**a**) Nyquist plots; (**b**) Bode impedance magnitude plots; (**c**) bode plots of phase angle vs. frequency, and the corresponding equivalent circuit modes (**d**).

Figure 8 displays the electrochemical impedance spectroscopy (EIS) measurements of AM60B coupled with DC01 after three hours in solutions with different NaCl concentrations. Figure 8a shows the Nyquist plot, while Figure 8b,c present the impedance and phase angle plots of the Bode diagram, respectively. In comparison to the EIS before coupling (Figure 6), the EIS after coupling incorporates an additional Q_p_ and R_p_ circuit in series with the circuit from Figure 6e to represent the corrosion product layer formed over the surface after corrosion, as well as the surface damage caused by corrosion. The fitted parameters are summarized in Table 5.

In the Nyquist plots after three hours of coupling, two capacitive loops and one inductive loop were observed under different concentration conditions. The two capacitive loops correspond to the mid-frequency and high-frequency regions, while the inductive loop corresponds to the low-frequency region. As the NaCl concentration increased, the diameter and modulus of the capacitive loops gradually decreased, and the impedance values in the Bode plots also decreased. Compared to the uncoupled state, the diameter of the capacitive loop after coupling was significantly smaller, with a difference in modulus of two orders of magnitude. These results indicated that coupling with DC01 caused severe degradation of the corrosion resistance on the surface of the AM60B alloy under different NaCl concentration conditions.

### 3.3. Galvanic Corrosion of AM60B/DC01

**Figure 9:**
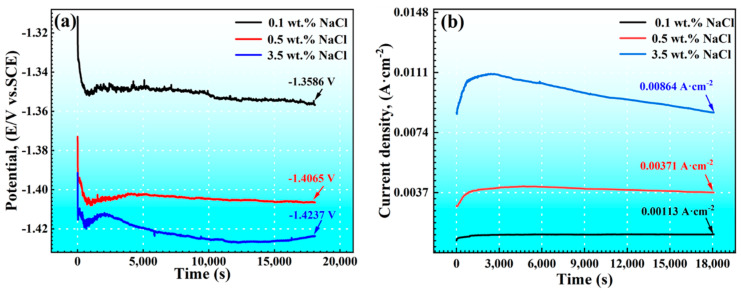
Time dependence of the coupled potential (**a**) and galvanic current density (**b**) between AM 60B and DC01 in 0.1 wt. %, 0.5 wt. %, and 3.5 wt. % NaCl solutions.

Figure 9 illustrates the curves of the galvanic corrosion potential and the current density as a function of time, obtained from different NaCl concentration conditions. In Figure 9a, the corrosion potential decreased with the increase in the NaCl concentration, while the corrosion current density increased with the NaCl concentration. This indicates that the higher NaCl concentration promotes the progress of corrosion. Similar fluctuations in the corrosion potential were observed in solutions with different NaCl concentrations. Specifically, the corrosion potential experiences a rapid decrease, followed by a slight reduction in the rate of decline during the initial stage of corrosion (within 10 min). Subsequently, a minor increase is observed, eventually reaching a stable state. The variation in corrosion potential over time provided insights into the specific process of galvanic corrosion. The sharp initial decrease in the corrosion potential was attributed to the breakdown of the corrosion products caused by the galvanic effect, resulting in surface damage. As the damaged area expanded, the corrosion potential exhibited a slight decrease. When the damage spreads across the entire surface, corrosion products cover the alloy surface, impeding further corrosion to some extent, leading to a minor increase in corrosion potential and eventually reaching a steady state. The corrosion potentials recorded under different concentration conditions at 3 h were −1.3586 V (0.1 wt. %), −1.4065 V (0.5 wt. %), and −1.4237 V (3.5 wt. %), respectively.

**Figure 10:**
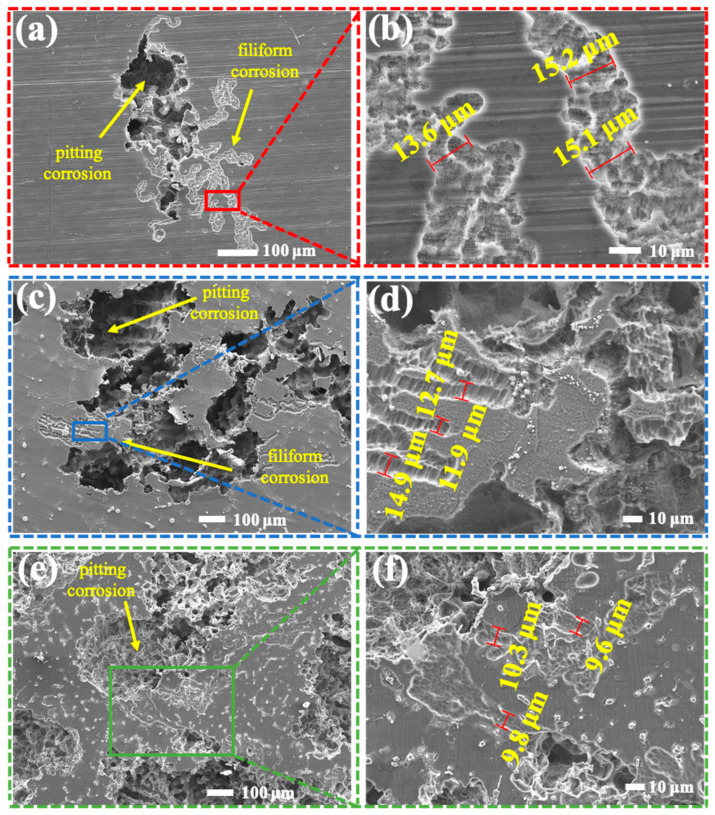
Morphologies of AM60B after removal corrosion products formed after 3 h exposure to: (**a**,**b**) 0.1 wt. %, (**c**,**d**) 0.5 wt. %, and (**e**,**f**) 3.5 wt. % NaCl solutions.

Figure 10 shows the morphologies and corresponding elemental surface distributions of AM60B after the removal of the corrosion products formed after a 3 h exposure to different NaCl concentrations. It can be observed that AM60B exhibited filament-like corrosion under different NaCl concentrations, but the morphology of the filament-like corrosion under different concentrations was different. Specifically: At 0.1 wt. % concentration, filamentary corrosion mainly occurred around pitting corrosion, and the depth and width of the filamentary corrosion were longer. When the concentration increased, the occurrence of filamentous corrosion increased, but the depth and width decreased with the increase in the NaCl concentration. Filamentous corrosion, also known as subfilm corrosion, mostly occurs under a protective coating, while, in uncoated magnesium alloy, impregnation bubbles can also cause filamentous corrosion in a NaCl solution [24]. Filament-like corrosion has a certain growth rule: there will be a long induction period before the occurrence of filament-like corrosion [25], and no filament-like corrosion will occur before the end of the induction period. Filament-like corrosion occurs in AM60B when it is coupled but is not found when it is not coupled, which indicates that the coupling with DC01 shortens the induction period of AM60B filament-like corrosion and promotes the initiation of filamentous corrosion.

### 3.4. FEM Modeling

**Figure 11:**
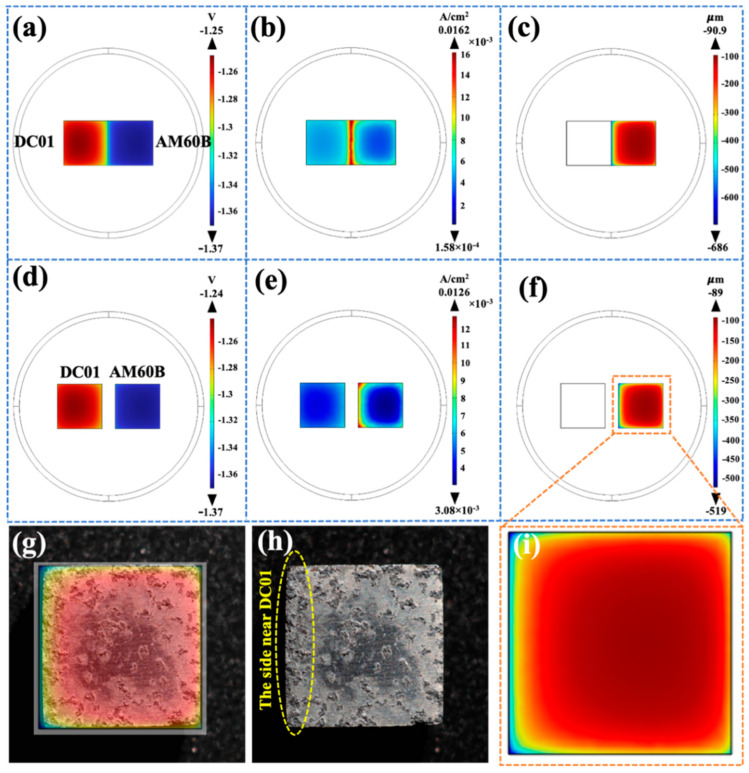
Effects of AM60B/DC01 coupling modes on galvanic corrosion: (**a**) electrode potential in direct contact state; (**b**) electrode current density in direct contact state; (**c**) corrosion depth of the electrode surface in direct contact; (**d**) electrode potential at wire connection; (**e**) electrode surface current density at wire connection; (**f**,**i**) electrode surface corrosion depth when wires are connected; (**g**) comparison of experimental data and simulation results; (**h**) corrosion morphology of AM60B after coupling with DC01 for three hours at 30 °C.

Figure 11 shows the influence of different coupling modes of AM60B/DC01 on galvanic corrosion. By comparison, it was found that the side of the two electrodes close to each other was slightly polarized when the wires were coupled (Figure 11b). When AM60B is in direct contact with DC01, the two metals have a greater degree of local polarization at the contact site. At the same time, Figure 11d,e show the electrode surface current density under two different coupling modes. It was found that, when AM60B is in direct contact with DC01, the current density at the contact site of the two electrodes was the highest (0.0162 A/cm^2^). When connected through a wire, the area with the highest current density was the side where the two electrodes were close to each other, and the maximum value was 0.0126 A/cm^2^, which is smaller than that when the electrodes were in direct contact. Figure 11c,f show the surface corrosion depth diagram of AM60B with two different coupling modes. The maximum corrosion depth of both coupling modes occurred at the side of the AM60B near the DC01, and the maximum corrosion depth was 686 µm with direct contact and 519 µm with a wire connection. The above results show that, no matter which coupling method was used, the most severely corroded area on the surface of AM60B was near DC01. By comparison with the above experiments (Figure 11g–i), it can be observed in Figure 11h that the corrosion is the least in the middle area of the surface of AM60B, while the corrosion near DC01 is the most serious. The experimental results are consistent with the simulation results.

**Figure 12:**
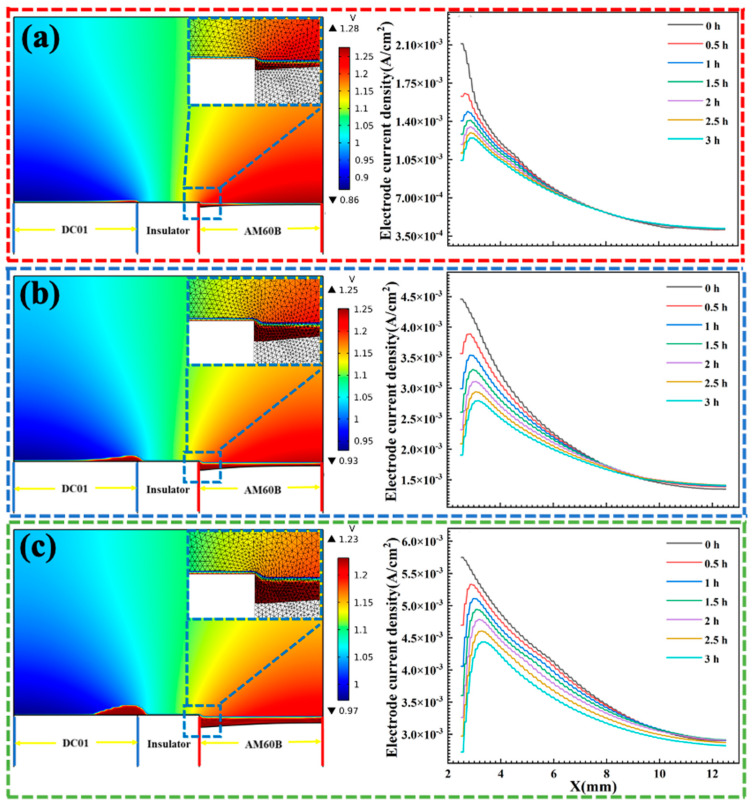
Galvanic corrosion potential distribution and electrode current density of AM60B/DC01 in solutions with different NaCl concentrations: (**a**) 0.1, (**b**) 0.5, and (**c**) 3.5wt. %.

Figure 12 shows the potential distribution of the galvanic corrosion electrolyte, deformation of anode electrode, deposition of corrosion products, and current density distribution of the AM60B electrode at different coupling times under different concentration conditions. The model follows charge conservation and local electro-neutrality, so the electrode surface potential distribution can be known according to the electrolyte potential distribution at the electrode interface, and only the electrolyte potential at the electrode interface is negative, which is the local electrode potential. As the NaCl concentration increased, the corrosion potential gradually decreased, indicating that the driving force of corrosion increases with the increase in the NaCl concentration. From the current density distribution diagram of the AM60B electrode on the right side, it can be found that the current density near DC01 is the highest, indicating that the corrosion rate in this area is the highest.

**Figure 13:**
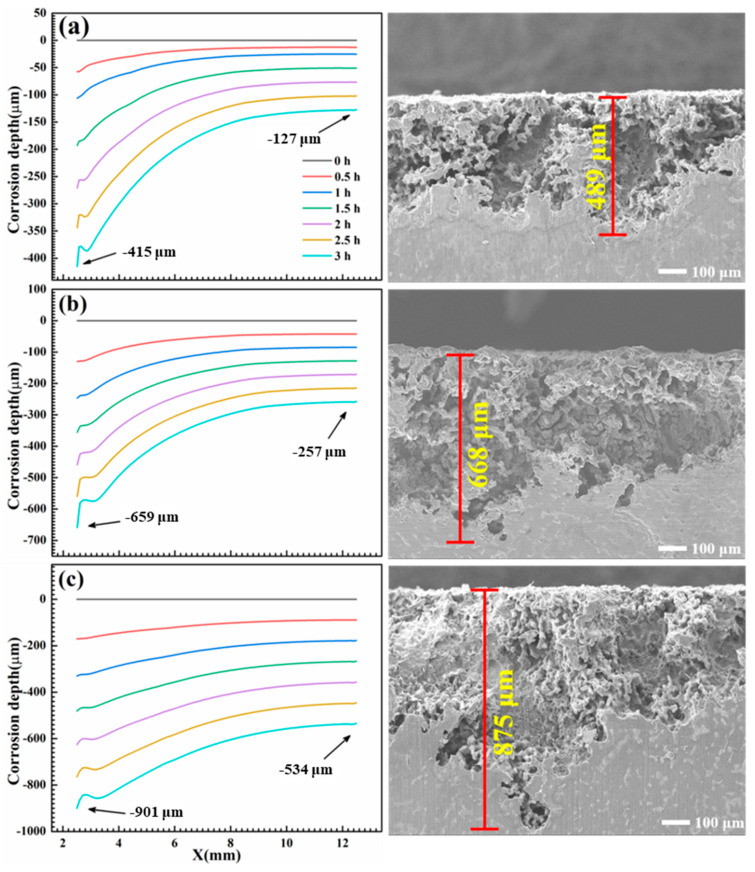
Anodic corrosion depth change of AM60B/DC01 within 3 h of galvanic corrosion: (**a**) 0.1, (**b**) 0.5, and (**c**) 3.5 wt. %.

Figure 13 shows the anodic corrosion depth variation of AM60B coupled to DC01 after immersion in 0.1, 0.5, and 3.5 wt. % NaCl solutions for 3 h, which was also proved by the fact that anodic AM60B had the deepest corrosion on the side close to the cathode DC01, while the corrosion depth was the shallowest on the side far away. The simulation results of corrosion depth were basically consistent with the experiment data, which proved that the increase in the NaCl concentration promoted the process of galvanic corrosion and also explained the reason why the formation of corrosion products was not limited to the corrosion area but covered the entire surface due to the ionic migration caused by the electric field during coupling.

### 3.5. Effect of DC01 on Galvanic Corrosion

As indicated, the coupled DC01 effectively accelerated the corrosion process of AM60B, and the higher salt concentration, the more obvious the acceleration effect of the electric couple was [26,27,28]. Generally, the potential difference dominates the possibility of galvanic corrosion between dissimilar materials in the conductive circuit, and corrosion current exhibits the corrosion rate [28,29,30]. And whether the materials acted as an anode or a cathode was decided by the combined action of the corrosion potential and the corrosion current. When coupled to DC01, the corrosion of AM60B was more serious than that of the uncoupled one. The corrosion potential of AM60B is much lower than that of DC01, and both of them have a similar corrosion current; therefore, AM60B acts as anode in the coupling system and exhibits serious corrosion [31,32,33,34]. Figure 11 and Figure 12 show the potential distribution of AM60B coupled to DC01 in NaCl solution of different concentrations. The electrolyte solution connects AM60B and DC01 in the conductive circuit. Due to the geometric features, the corrosion depth presents an obvious corner effect; that is, the corrosion degree near DC01 is more serious. At the same time, the increase in salt concentration also exacerbated the process of galvanic corrosion due to the better conductivity in the closed circuit (the maximum corrosion depth of AM60B at 0.1, 0.5, and 3.5 wt. % was 0.489, 0.668, and 0.875 mm, respectively).

Based on the above experimental results and analysis, the corrosion process of AM60B in the coupling process was obtained. In the initial stage, the entire surface of AM60B is involved in an electrochemical reaction dominated by metal dissolution, and the corrosion product Mg(OH)_2_ is widely generated and dissolved in large quantities into the electrolyte solution. Usually this process is short, perhaps only a few minutes, but it can restore the potential of AM60B to the initial state of bare metal. Then, the corrosion products only cover the local area of AM60B, showing the characteristics of local corrosion [35,36,37,38,39] (as shown in Figure 12). The following chemical reactions occur on the anode and cathode [39,40,41]:

Mg – 2e^−^ → Mg^2+^ (Anode) (3)

H_2_O + 1/2O_2_ + 2e^−^ → 2OH^−^ + H_2_↑ (Cathode) (4)

Mg^2+^ + 2OH^−^ → Mg(OH)_2_↓ (Anode) (5)

Reaction (4) mainly occurs on the cathode DC01, releasing H_2_. The reticulated β-Mg_11_Al_12_ phase has distinct potential difference with the α-Mg matrix, which can form a micro-couple [36,37,38,39,40]. Since the potential of the β-Mg_11_Al_12_ phase is significantly higher than that of the α-Mg matrix, the β-Mg_11_Al_12_ phase is usually acts as anode and corrodes the α-Mg matrix. In addition, Mg(OH)_2_ is loose and easily forms water-soluble corrosion products, and its protective properties are weak, making it difficult to form a physical shielding [38,39,40,41]. The acceleration effect of the coupled DC01 electrode on AM60B is mainly reflected in accelerating the dissolution of AM60B, while the influence of the AM60B surface corrosion products on the galvanic corrosion is weak [41,42].

## 4. Conclusions

The galvanic corrosion behavior of AM60B alloy coupled to DC01 was assessed in various NaCl solutions to simulate the various saltwater environments. Results showed that the coupled DC01 effectively accelerated the corrosion rate of AM60B, and the increased salt concentration had a significant effect on the deterioration of its corrosion. The corrosion of AM60B is mainly based on metal dissolution, and the corrosion products have a weak shielding effect on the surface, which lacks effective ability to prevent the reaction. Furthermore, the existence of macroscopic coupled DC01 annihilates the micro-potential difference between α and β inside the AM60B, and the interphase galvanic corrosion is replaced by entire corrosion.

## Figures and Tables

**Table 1:** Tafel fitting parameters of polarization curves of AM60B in 0.1, 0.5, and 3.5 wt. % NaCl solutions.

NaCl	E_corr_ (V)	i_corr_ (μA/cm^2^)	β_a_ (V/dec)	β_c_ (V/dec)
0.1 wt. %	−1.38	1.62	0.13	−0.09
0.5 wt. %	−1.49	8.61	0.03	−0.12
3.5 wt. %	−1.54	57.94	0.04	−0.10

**Table 2:** Tafel fitting parameters of the polarization curves of DC01 in 0.1, 0.5, and 3.5 wt. % NaCl solutions.

NaCl	E_corr_ (V)	i_corr_ (μA/cm^2^)	β_a_ (V/dec)	β_c_ (V/dec)
0.1 wt. %	−0.52	6.59	0.04	−0.06
0.5 wt. %	−0.61	7.45	0.08	−0.15
3.5 wt. %	−0.67	9.13	0.06	−0.06

**Table 3:** Impedance spectroscopy equivalent circuit fitting parameters of AM60B in 0.1, 0.5, and 3.5 wt. % NaCl solutions.

NaCl	R_S_ (Ω·cm^2^)	Q_f_ (F·cm^−2^)	n	R_f_ (Ω·cm^2^)	Q_dl_ (F·cm^−2^)	n	R_ct_ (Ω·cm^2^)	L (H)	R_L_ (Ω·cm^2^)
0.1 wt. %	212	1.12 × 10^−5^	0.92	3.40 × 10^3^	9.53 × 10^−4^	0.80	2640	—	—
0.5 wt. %	44	9.91 × 10^−6^	0.94	1.24 × 10^3^	7.14 × 10^−5^	0.99	265	4.45 × 10^4^	8.44 × 10^3^
3.5 wt. %	6	1.04 × 10^−5^	0.94	5.36 × 10^2^	9.13 × 10^−5^	0.99	288	4.95 × 10^3^	2.17 × 10^3^

**Table 4:** Equivalent circuit fitting parameters of impedance spectra for DC01 in 0.1, 0.5, and 3.5 wt. % NaCl solutions.

Condition	R_S_ (Ω·cm^2^)	Q_f_ (F·cm^−2^)	n	R_f_ (Ω·cm^2^)	Q_dl_ (F·cm^−2^)	n	R_ct_ (Ω·cm^2^)
0.1 wt. %	173	9.72 × 10^−4^	0.68	1177	1.09 × 10^−3^	0.46	1298
0.5 wt. %	28	1.35 × 10^−3^	0.67	65	1.39 × 10^−4^	0.99	1139
3.5 wt. %	6	1.58 × 10^−3^	0.67	405	2.68 × 10^−3^	0.45	535

**Table 5:** Equivalent circuit fitting parameters of impedance spectra of AM60B coupled with DC01 for three hours in 0.1, 0.5, and 3.5 wt. % NaCl solutions.

Condition	R_S_ (Ω·cm^2^)	Q_f_ (F/cm^2^)	n_2_	R_f_ (Ω·cm^2^)	Q_dl_ (F/cm^2^)	n_3_	R_ct_ (Ω·cm^2^)	L(H)	R (Ω·cm^2^)
0.1 wt. %	45	1.36 × 10^−7^	0.39	229	3.07 × 10^−4^	0.99	210	6.99 × 10^3^	1512
0.5 wt. %	34	4.52 × 10^−5^	0.99	185	2.20 × 10^−5^	0.80	69	1.46 × 10^3^	388
3.5 wt. %	32	3.06 × 10^−5^	0.99	302	9.70 × 10^−5^	0.59	73	3.83 × 10^3^	105

## Data Availability

The original contributions presented in the study are included in the article, further inquiries can be directed to the corresponding author.
